# Perceptions and attitudes towards dementia among university students in Malaysia

**DOI:** 10.1186/s12909-020-1972-5

**Published:** 2020-03-20

**Authors:** Alys Wyn Griffiths, Wing Loong Cheong, Pui San Saw, Sahdia Parveen

**Affiliations:** 1 Centre for Dementia Research, Leeds Beckett University, Leeds, UK; 2 School of Pharmacy, Monash University Malaysia, Subang Jaya, Malaysia; 3 Centre for Applied Dementia Studies, University of Bradford, Bradford, UK

## Abstract

**Background:**

One of the major challenges worldwide is the stigma associated with dementia. There is limited dementia awareness within Malaysian communities, including levels of confusion regarding the differences between dementia and the usual ageing progress, which can lead to delays in support seeking. The need for additional training and education for healthcare professionals has been highlighted. The present study aimed to evaluate the benefits of a one-hour dementia education session (Dementia Detectives workshop) for pharmacy and medicine undergraduate students at a Malaysian university.

**Methods:**

Participants attended the workshop and completed pre- (Time 1) and post-workshop (Time 2) questionnaires consisting of validated measures exploring attitudes towards dementia and older people more broadly.

**Results:**

A total of 97 students were recruited. Attitudes towards people with dementia showed significant positive changes between Time 1 and Time 2, whereas no differences were found for attitudes towards older people.

**Conclusions:**

As medical and pharmacy students develop theoretical knowledge, practical skills and professional attitudes during their undergraduate studies, it is important for students to also learn about the humanistic side of diseases and conditions through workshops such as the one presented here. Further research should now be conducted to consider how Dementia Detectives can be delivered to non-healthcare students and what the barriers and facilitators to wider delivery are.

## Background

Dementia is the largest challenge for health and social care worldwide [18]. Rates of dementia are increasing globally, with the largest growth in numbers expected amongst those living in low-and middle-income countries (LMICs [26];). In 2010, 58% of people with dementia resided in LMICs and this is set to rise to 63% by 2030, and 71% by 2050 [26]. In 2005, the number of people with dementia in Malaysia, a World Bank high middle-income country, was estimated at 63,000 with 20,100 incident cases per annum. This is projected to increase to 126,800, with 39,000 annual cases by 2020 [1], although this is likely to be an underestimate due to issues with inadequate assessment and diagnosis [30]. Additionally, there is an aging population in Malaysia, with 15% expected to be aged 65 or over by 2040 [11].

There are concerns about whether Malaysia is able to provide quality health and social care to support people with dementia [21], and broader concerns regarding how prepared Malaysian families are to care for relatives with dementia [2], as most experience moderate burden when caring for a relative with dementia [9]. Around a third of caregivers receive support from paid professionals and a similar amount receive support from other family members [9]. Dementia awareness is limited within Malaysian communities, including confusion regarding the differences between dementia and the usual ageing progress, which can lead to delays in seeking support or medical intervention [2]. For example, of 347 older adults attending a hospital in urban Malaysia for physical health conditions, the majority were found to have mild (64.7%) or moderate to severe (3.1%) cognitive impairment [27]. Furthermore, there is a lack of psychiatric and social support available for people with dementia in Malaysia, particularly those who live in rural areas [21].

One of the major challenges worldwide is the stigma associated with dementia. Negative attitudes and stigma towards people with dementia have been highlighted as issues in Malaysia [21]. There are four major factors that contribute to the stigma around mental illness [20]. These are that people with mental illness are dangerous; are personally responsible for succumbing to their illness; have a poor prognosis, and that the illness causes a severe disruption of normal social interaction. Despite dementia being a neurological condition, it is often met with the same fear and misunderstanding as mental illness [24].

Recognition of the impact of stigma on people with dementia and their families has increased [4] and has led to many countries developing and implementing National Dementia Strategies (NDS). Among the 29 countries with a NDS, increasing dementia awareness and reducing stigma were common priorities, with plans to achieve this mainly focusing on awareness campaigns, supplemented by educational initiatives and interventions to reduce stigma [10]. However, despite neighbouring countries having a developed NDS, Malaysia does not currently have one in place. Additionally, there is currently no defined curriculum for dementia education in either schools or universities. To date, there has been no evaluation of any education delivery within the country for healthcare students, despite specialised dementia education and training being recommended across the Asia Pacific region [2]. This is recommended as students have been shown to have unfounded negative beliefs and stigma, that led to stereotypes and prejudice towards people living with the condition [20]. One Malaysian survey identified that qualified pharmacists had ‘moderate knowledge’ about dementia, but highlighted the need for additional training and education [22]. Younger participants, who had completed their education more recently, had higher dementia awareness, suggesting that there may be more dementia related content delivered in undergraduate programmes than there previously was [22]. A comparison of UK and Malaysia based medical students demonstrated more positive attitudes towards dementia amongst UK based students, with those in years 3 and 5 of their degrees showing the most positive attitudes [29]. To date, the impact of a specific educational session on attitudes towards dementia of healthcare students has not been considered. To ensure that a person-centred approach is provided across healthcare settings, this should include not only the biological aspects of dementia, but also the more ‘humanistic’ side, focusing on how dementia affects peoples’ lives.

The present study aimed to examine the benefits of a one-off dementia educational session on attitudes towards and knowledge of dementia amongst healthcare undergraduate students in Malaysia, using pre- and post-workshop questionnaires.

## Method

### Participants

Undergraduate students were recruited from the School of Pharmacy and School of Medicine at a Malaysian University to attend a one-hour dementia education workshop. All students studying in either school at Bachelors level were eligible to participate. Interested students signed up to attend a workshop online.

### Procedure

Participants were recruited to attend a dementia workshop and completed questionnaires before and after the workshop. Workshops were advertised via email, as well as recruitment posts on the student societies’ social media pages.

Participants attended a ‘Dementia Detectives’ workshop. This is a one-hour workshop, originally developed as an awareness initiative for secondary school students aged from 14 to 16 years [25] and recently adapted for university contexts in the UK (see [24] for description of workshop content). The workshop aims to increase understanding of dementia and develop positive person-centred attitudes towards those living with dementia. The detective theme is incorporated into the workshop as it centres on dispelling the myths surrounding dementia and key messages. Facilitators deliver the workshop (in the present study, AG, MC, and SPS), which involves presentation of content and interactive activities completed in five teams. There is no assessment or evaluation of participants within the workshop. Activities in the workshop include watching a video of a person with Alzheimer’s disease and discussing their thoughts on this, decoding messages to reveal key messages such as ‘dementia is not the same as ageing’, and writing down what they think of when they hear the word dementia (providing facilitators with specific misconceptions that can be addressed throughout the workshop). Additionally, there is a case study of a fictional person living with dementia who is described as behaving aggressively. Each team is given a clue, which represents one component of the biopsychosocial approach to dementia. After discussion, participants are presented with the full biopsychosocial model to demonstrate that behaviour may not be due to dementia alone but that neurological impairment interacts with a range of other factors.

Prior to the workshop being delivered, adaptations were made by the research team, which included the original developer of the workshop, to ensure that the content was culturally appropriate. This included changing case studies to represent the population of Malaysia in ways such as names, family roles and activities.

An electronic link to the questionnaire was emailed to all participants a week before they participated in the workshop (Time 1). They were asked to complete the same questionnaire immediately following the workshop (Time 2). A unique code, specified by the participant, was used to link their responses and ensured their anonymity was protected.

### Ethics, consent and permissions

Prior to the study being conducted, ethical approval was received from [redacted] University and [redacted] University. All participants provided informed written consent before participation.

### Measures

Participants completed several measures at both time points. Firstly, participants were asked to if they had heard of dementia and if they would like to learn more about dementia.

The Adolescent Level of Contact Scale (ALoCS [23];) consists of 10 items. In the present study, the internal consistency of the scale was .84 at Time 1 and .87 at Time 2. The scale assesses quantity and quality of direct and indirect (via the media) contact with people living with dementia. Each item is measured on a 5 point Likert scale.

The Adolescent Attitudes towards Dementia Scale (AADS [13];) is a 23-item validated measure of attitudes and knowledge of dementia, designed for use with adolescents. The measure covers 3 subdomains; perceptions of dementia, personal sacrifice, and empathy with people living with dementia. Items are rated on a 5-point Likert scale ranging from ‘strongly disagree’ to ‘strongly agree’. In the present study, the internal consistency of the scale was .73 at Time 1 and .81 at Time 2.

The Attitudes Towards Older People Scale (ATOP [17];) is a 34-item validated scale with 17 positive and 17 negative items. The scale covers three domains: personal appearance, resemblance, and the nature of interpersonal relations across age generations. In the present study, the internal consistency of the scale was .73 at Time 1 and .79 at Time 2.

The Illness Perceptions Questionnaire (IPQ [19];) is a 9-item validated measure that measures the following: perceptions of illness identity (symptoms), perceived timeline, consequences of illness, personal and treatment control, concern, cause of illness, coherence (understanding of illness) and emotional representations. The scale has been adapted for us with a wide range of illnesses and disorders [7]. In the present study, the brief IPQ was utilised to assess perceptions of dementia. The Brief IPQ [6] consists of one item to measure each illness perception on a 10 point Likert scale. The ‘Cause’ Scale involves participants listing three most important factors in causing dementia for example ‘head injury’.

## Results

### Preliminary analyses

A total of 125 participants registered to attend the workshops. Of these, 112 attended and completed one questionnaire, and 97 attended and completed both questionnaires. These 97 participants were included in the analyses presented here.

Most participants were female (*N* = 82; 84%) and described their ethnicity as Chinese (*N* = 88; 91%). Participant ages ranged from 16 to 24 (see Table 1 for participant demographics). Nearly all participants stated that they had heard of dementia previously (*N* = 93; 96%) and wanted to learn more about dementia (*N* = 91; 94%).

**Table 1:** Participant demographics

	N (%)
Gender	
Female	82 (84)
Male	15 (16)
Ethnicity	
Chinese	88 (91)
Indian	4 (4)
Sinhalese	1 (1)
Malay	1 (1)
Sikh	1 (1)
Sri Lankan	1 (1)
Egyptian	1 (1)
Age	
16	1 (1)
18	4 (4)
19	8 (8)
20	17 (18)
21	31 (32)
22	20 (21)
23	11 (11)
24	5 (5)
Course	
Pharmacy	81 (83)
Medicine	16 (17)

### Level of contact

The Adolescent Level of Contact scale was used to determine how much contact participants had previously had with people with dementia. Generally, participants had a moderate level of indirect contact (M = 13.13, SD = 4.07) and low level of direct contact (M = 8.03, SD = 4.64) with people with dementia. The most frequent ways that participants had experienced contact were through watching TV shows/movies (*N* = 90; 93%), searching for information on the internet (*N* = 86; 89%) and adverts within the community (*N* = 79; 81%).

### Changes in attitudes and perceptions

To understand whether there were changes in attitudes and knowledge towards dementia following the workshop, paired sample t-tests were conducted.

Positive attitudes towards people with dementia (measured by the AADS) showed significant increase between Time 1 and Time 2 (t(96) = 8.14, *p* < .001). Time 1 scores ranged from 63 to 103 (M = 87.18, SD = 7.12) and Time 2 scores ranged from 75 to 110 (M = 92.81, SD = 7.81).

There were no differences in attitudes towards older people in general (measured by ATOP) between Time 1 and Time 2 (t(96) = .19, *p* > .05). Time 1 scores ranged from 102 to 158 (M = 123.39, SD = 11.65) and Time 2 scores ranged from 96 to 162 (M = 123.28, SD = 12.97).

**Fig. 1:**
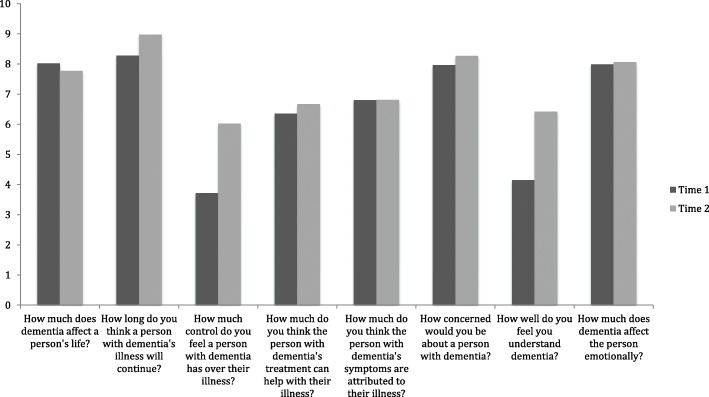
Changes in perceptions of dementia between Time 1 and Time 2

Perceptions of dementia varied between the two time points (see Fig. 1). Significant increases in perceptions of the length of time dementia affects someone’s life (t(96) 3.828, *p* < .001), the control someone with dementia has over their illness (t(96) 4.09, p < .001), level of concern about a person with dementia (t(96) 2.09, *p* < .05), and how well participants understand dementia (t(96) 10.50, *p* < .001). Nonsignificant changes were found for the remaining items.

At Time 1, 12 participants stated that they did not know any causes of dementia, whereas at Time 2 all participants were able to provide at least one cause. Interestingly at time 1, 18% of participants identified old age as a cause of dementia and this reduced to 4% at time 2. Approximately 33% of participants were able to correctly identify potential risk factors for dementia at time 1, and this increased to 49% after the workshop.

## Discussion

The current study provides the first evidence of the effectiveness of a dementia awareness workshop on attitudes towards and perceptions of dementia amongst undergraduate and postgraduate students in Malaysia.

Participants generally held positive attitudes towards people with dementia. As medical and pharmacy students, they spend a large proportion of their curriculum in clinical environments and are expected to learn theoretical knowledge, practical skills and professional attitudes during these placements. It is therefore important for students to also learn the humanistic side of diseases and conditions [5] and be able to integrate scientific findings and practical experience as they become healthcare professionals [16]. As stigma reduces the likelihood of people seeking a dementia diagnosis and subsequent post-diagnostic support [8], these results are encouraging from healthcare students. By encouraging the development of positive attitudes, students’ confidence and comfort in interacting with people with dementia can be improved [5]. As the number of people with dementia continues to rise, it is necessary to prepare and to ensure that future members of the healthcare workforce are able to recognise, manage, and support the complex needs of people living with dementia.

However, no differences were found in participants’ attitudes towards older people. One of the key messages of the workshop is ‘dementia is not a natural part of ageing’ and a video within the workshop shows a younger person with dementia. Therefore it may be that participants were not considering ‘older people’ as a population during the workshop and therefore there was no impact on their attitudes towards this group. This should be explored further using qualitative methods to help understand how the workshop impacts on participants’ attitudes.

The level of contact that participants had experienced with people with dementia previously varied, in line with existing research. In Australia, 31% of adolescents stated that they had never seen a person with dementia other than in passing, and 24% stated that they knew either a friend or family member with dementia [3]. Additionally, 23% of adolescents in England reported knowing someone with dementia [15]. More recently, 37% of participants stated that they had spent time with a family member with dementia [12]. Perhaps more interestingly, many more participants have seen a TV programme, film, news story, or advert about dementia than know someone in dementia in both England (80%, [12]) and Australia (37% [3];). This may highlight the growth in presence of dementia in the media, or may represent differences between the countries. As mass media campaigns are a successful way of distributing health related messages, this may be an appropriate for Malaysia, and is currently being trialled for the communication of cancer related information [28].

Increasing levels of contact with people with dementia is key in reducing stigma [20] and therefore any interventions developed to increase awareness and understanding of dementia amongst young people in Malaysia need to ensure the involvement of people with dementia in the development and delivery.

Previous evidence has highlighted the need for additional training and education for healthcare students around dementia [22]. Though Dementia Detectives was conducted as a short workshop, it has shown a positive impact on the attitudes and perceptions of students. This further supports the future use of the workshop as an educational tool since many students in Malaysia may not receive any formal education about dementia. Therefore, incorporating this into the curriculum of healthcare courses would ensure that students have a fundamental understanding of dementia while paving the way for the development of more advanced courses on supporting people living with dementia. This is particularly important as medical students in Malaysia have less positive attitudes towards dementia than UK based students [29], where dementia is included in the majority of healthcare courses and there is a specific dementia education and training framework [14] developed from nationwide policy. Without specific education focusing on reducing stigma and misunderstanding about dementia, attitudes towards the condition cannot be changed. Dementia education should be incorporated into the curriculum on a national level in Malaysia, to ensure that future generations are informed about, and prepared for living with dementia. Future research should establish the optimal delivery method and student age for dementia education.

Currently there is no National Dementia Strategy in Malaysia. As limited dementia awareness, and negative attitudes and stigma towards people with dementia have been highlighted as issues in Malaysia [21], the Dementia Detectives workshop offers an opportunity to address these issues in the short term, whilst a strategy is developed. Additionally, a dementia education and training framework should be developed to ensure that future healthcare professionals receive appropriate training to support people living with dementia.

There are several limitations associated with the present study. Firstly, all participants were recruited from medicine and pharmacy courses within a single university. We did not collect any information on whether participants had previously received dementia education within their courses. Therefore, these findings may not represent the views held by students more widely, particularly those students who are not studying health sciences. Future research will need to explore if this is unique to the courses identified in this study and if not, the effective ways to improve attitudes towards dementia among students in general. Additionally, participants in this study opted in to participate, rather than the workshop being delivered as part of their curriculum. Therefore, it may be that these individuals were generally interested in learning about dementia and held more positive attitudes towards and perceptions of dementia, and those with less positive attitudes did not sign up to attend the workshop. Participants completed the second questionnaire within 24 h of participating in the workshop. Therefore, we do not know whether the positive effects on attitudes and perceptions found in this study are maintained over time. However, within the present study we aimed to establish whether the workshop had any impact on these factors, rather than to establish whether this potential impact was maintained. Future research should include an additional longer follow-up period to understand how attitudes change over time after attending the workshop. Additionally, this should include more in-depth analysis to explore whether any changes in attitudes observed are moderated or mediated by other factors, such as level of contact with people with dementia prior to receiving education.

Future research should examine the impact of the Dementia Detectives workshop on students studying non-healthcare related subjects. These students would be expected to have less exposure to dementia and therefore may not show neutral or positive attitudes towards dementia at baseline. Furthermore, work should be conducted with young people to establish what they believe the core components of a dementia education workshop should look like, and establish the barriers and facilitators to delivering Dementia Detectives more widely. Similar work in the UK highlighted that students were interested in learning a range of information about dementia, but perceived that they would benefit from the inclusion of a person with dementia delivering content [24].

## Conclusions

In conclusion, this study highlights that whilst some students held positive attitudes towards and perceptions of dementia, there are also some who do not share these views. We found that one-hour Dementia Detectives workshop is associated with improved attitudes towards and perceptions of dementia for Malaysian students. Further research should now be conducted to consider how Dementia Detectives can be delivered to non-healthcare students and what the barriers and facilitators to wider delivery are.

## Data Availability

The datasets generated and/or analysed during the current study are not publicly available due to ethics approval but are available from the corresponding author on reasonable request.

## Abbreviations

AADS: Adolescent attitudes towards dementia scale
ALoCS: Adolescent level of contact scale
ATOP: Attitudes towards older people scale
IPQ: Illness perceptions questionnaire
NDS: National dementia strategy
